# White-tailed deer are susceptible to the agent of sheep scrapie by intracerebral inoculation

**DOI:** 10.1186/1297-9716-42-107

**Published:** 2011-10-11

**Authors:** Justin J Greenlee, Jodi D Smith, Robert A Kunkle

**Affiliations:** 1 Virus and Prion Research Unit, National Animal Disease Center, ARS, USDA, Ames, IA 50010, USA

## Abstract

Interspecies transmission studies afford the opportunity to better understand the potential host range and origins of prion diseases. The purpose of this experiment was to determine susceptibility of white-tailed deer to the agent of scrapie after intracerebral inoculation and to compare clinical signs and lesions to those reported for chronic wasting disease (CWD). Deer (*n *= 5) were inoculated with 1 mL of a 10% (wt/vol) brain homogenate derived from a sheep clinically affected with scrapie. A non-inoculated deer was maintained as a negative control. Deer were observed daily for clinical signs of disease and euthanized and necropsied when unequivocal signs of scrapie were noted. One animal died 7 months post inoculation (pi) due to intercurrent disease. Examinations of brain tissue for the presence of the disease-associated abnormal prion protein (PrP^Sc^) by western blot (WB) and immunohistochemistry (IHC) were negative whereas IHC of lymphoid tissues was positive. Deer necropsied at 15-22 months pi were positive for scrapie by IHC and WB. Deer necropsied after 20 months pi had clinical signs of depression and progressive weight loss. Tissues with PrP^Sc ^immunoreactivity included brain (at levels of cerebrum, hippocampus, colliculus, cerebellum, and brainstem), trigeminal ganglion, neurohypophysis, retina, spinal cord, and various lymphoid tissues including tonsil, retropharyngeal and mesenteric lymph nodes, Peyer's patches, and spleen. This work demonstrates for the first time that white-tailed deer are susceptible to sheep scrapie by intracerebral inoculation. To further test the susceptibility of white-tailed deer to scrapie these experiments will be repeated with a more natural route of inoculation.

## Introduction

Scrapie is the naturally-occurring prion disease of sheep and goats. Chronic wasting disease (CWD) is the naturally-occurring prion disease of cervids and occurs in free-ranging herds of North America [1] and captive cervids in North America [2,3] and Korea [4,5]. While scrapie has been known for hundreds of years [6], chronic wasting disease of deer and elk has been relatively more recently described [2,3]. Scrapie and CWD in their respective hosts share similarities including widespread accumulation of abnormal prion protein (PrP^Sc^) in lymphoid and nervous tissues [7-9].

The first known occurrence of CWD was in captive populations, but the original source of the disease remains unknown. CWD has been hypothesized to be derived from scrapie [10]. Previous publications describing similar western blot profiles of tissues from cervids with CWD and sheep with scrapie [11] and in vitro conversion assay results indicating PrP compatibility between sheep and deer [12] lend further support to this theory. In previous studies, intracerebral inoculation of elk with brain homogenate from scrapie-affected sheep caused a disease similar to scrapie and was indistinguishable from CWD by microscopic examination or immunohistochemistry [13,14].

Interspecies transmission studies afford the opportunity to better understand the potential host range and origins of prion diseases. Because it is possible for wild cervids to live on the same range as sheep or to contact sheep at domestic/wildlife interfaces in many parts of North America, it is important to understand whether scrapie could transmit to cervids if exposed. The purpose of this experiment was to determine the susceptibility of white-tailed deer to the agent of sheep scrapie after intracerebral inoculation and to compare clinical signs and lesions to those reported for chronic wasting disease (CWD). This study documents that each of the inoculated deer had evidence of PrP^Sc ^accumulation and details histopathologic, immunohistochemical, and western blot (WB) findings.

## Materials and methods

All animal experiments described were reviewed and approved by the National Animal Disease Center's Institutional Animal Care and Use Committee (protocol number 3804) and were carried out in strict accordance with the Guide for the Care and Use of Laboratory Animals (Institute of Laboratory Animal Resources, National Academy of Sciences, Washington, DC) and the Guide for the Care and Use of Agricultural Animals in Research and Teaching (Federation of Animal Science Societies, Champaign, IL). White-tailed deer fawns were obtained from a farm that has never had a case of chronic wasting disease and is located outside of a CWD endemic area. Deer were inoculated intracerebrally with 1 mL of inoculum (*n *= 5) or were maintained as non-inoculated controls (*n *= 1). Inoculated deer were housed in a biosafety level-2 containment facility. The non-inoculated deer was housed in a separate location with other non-inoculated deer. Deer were observed daily for clinical signs of disease and euthanized and necropsied when unequivocal signs of TSE were noted or when euthanasia was necessary due to intercurrent illness or injury.

The inoculum (#13-7) [7] was a 10% (w/v) homogenate in phosphate-buffered saline with 100 μg/mL gentamicin that had been passaged four times through susceptible sheep that were homozygous ARQ at residues 136, 154, and 171, respectively [15]. The procedure for intracerebral inoculation has been described previously [7]. Briefly, the animals were sedated with xylazine, the frontal area was clipped and scrubbed, a 1 cm midline incision was made in the skin slightly caudal to the junction of the parietal and frontal bones, and a 1-mm hole was drilled through the calvarium. A 22-guage spinal needle was advanced through the hole perpendicular to the frontal bones until the tip of the needle made contact with the opposite (bottom) side of the calvarium. The inoculum was slowly injected as the needle was withdrawn through the brain. The skin was closed with a single suture.

At necropsy two sets of tissue samples including representative sections of liver, kidney, spleen, skin, striated muscles (heart, tongue, diaphragm, masseter), thoracic aorta, thyroid gland, turbinates, trachea, lung, tonsils, esophagus, rumen, reticulum, omasum, abomasum, intestines (ileum), adrenal gland, urinary bladder, lymph nodes (retropharyngeal, prescapular, mesenteric, popliteal), nerves (sciatic, optic, trigeminal), pituitary gland, trigeminal ganglion, brain (cerebral cortex, cerebellum, midbrain including superior colliculus, brainstem including obex), spinal cord (cervical, thoracic, lumbar), and eye were collected. The first set was collected into 10% buffered formalin (globes were fixed in Bouin's fixative), embedded in paraffin wax, and sectioned at 5 μm for staining with hematoxylin and eosin (HE) and anti-prion protein antibodies. The second set of tissues was frozen.

Vacuolation profiles were generated by examining defined regions of brain on hematoxylin and eosin stained sections and scoring the level of vacuolation. The scrapie-inoculated deer of this study were compared to white-tailed deer from a prior study [16] inoculated intracerebrally with CWD derived from either white-tailed deer or elk. Gray matter regions evaluated included the rostral cerebral cortex, hippocampus, caudal colliculus, nodulus and flocculus of the cerebellum, and the parasympathetic nucleus of the vagus nerve, motor nucleus of the hypoglossal nerve, and reticular formation at the level of the obex. White matter was evaluated in the rostral cerebrum and cerebellum. For gray matter, scores were assigned as follows: 0 = no vacuolation; 1 = occasional vacuole indicative of aging or spongiform encephalopathy (SE), but diagnosis inconclusive; 2 = definitive SE defined as the presence of crisp, round to oval vacuoles within neurons and/or neuropil, but no even spread of vacuoles; 3 = even spread of vacuoles throughout the region with coalescence of some vacuoles; 4 = heavy, even spread of vacuoles throughout the region with frequent coalescence (most severe). For white matter: 0 = no definite SE or occasional inconclusive axonal vacuolation, 1 = definite SE, but no even spread; 2 = even spread of SE throughout the region; 3 = heavy, even spread of SE throughout the region with frequent coalescence (most severe). Scores were averaged for each group of deer and graphed using standard data management software (Excel for Mac 2008, Microsoft, Redmond, WA, USA).

All paraffin-embedded tissues were also stained by an automated immunohistochemical method for detection of PrP^Sc ^as described previously [7] with slight modifications. Briefly, after deparaffinization and rehydration, tissue sections were autoclaved for 30 min in an antigen retrieval solution (DAKO Target Retrieval Solution, DAKO Corp., Carpinteria, CA, USA) and stained with an indirect, biotin-free staining system containing an alkaline phosphatase labeled secondary antibody (*ultra*view Universal Alkaline Phosphatase Red Detection Kit, Ventana Medical Systems, Inc., Tucson, AZ, USA) designed for an automated immunostainer (NexES IHC module, Ventana Medical Systems, Inc., Tucson, AZ, USA). The primary antibody used was F99/97.6.1 [17] at a concentration of 5 μg/mL, and incubation was carried out at 37°C for 32 min. Images for the figures were captured using a Nikon DS camera on a Nikon Eclipse 80*i *microscope.

Frozen brain tissues were used for immunodetection of PrP^Sc ^by WB. Brain samples from the brainstem (at the level of obex) and cerebrum were homogenized at a final concentration of 10% (w/v) in 1× homogenization buffer (Prionics AG, Switzerland) using a tissue homogenizer (Powergen 125 homogenizer, Fisher Scientific) with disposable probe. An equal volume of 20% N-laurylsarcosine in 10 mM Tris pH 7.5 was then added to the homogenates, and the samples were vortexed for 30 min at room temperature and then centrifuged at 10 000 × *g *at 10°C for 30 min. The supernatants were collected and ultracentrifuged at 186 000 × *g *for 55 min at 10°C. The pellets were then resuspended in 100 μL of water. Proteinase K (USB, Cleveland, OH, USA) was added to a final concentration of 8.0 μg/mL and samples were incubated at 37°C for 30 min. The reactions were terminated by the addition of phenylmethanesulfonylfluoride to a final concentration of 5 mM and incubated on ice for 15 min. Samples were then ultracentrifuged at 186 000 × *g*, and the pellets resuspended in water to 1-5 mg tissue equivalents/μL. SDS Gel sample buffer (Biorad Laboratories, Hercules, CA, USA) (4×) was added to a final concentration of 1× and samples were loaded onto a 12% commercially prepared SDS-PAGE gel (Invitrogen, Carlsbad, CA, USA) at ~1 mg tissues equivalent in SDS-PAGE sample buffer and run according to the manufacturer's instructions, blotted to a polyvinylidene difluoride (PVDF) membrane (GE Healthcare, Buckinghamshire, UK) and blocked with 3% bovine serum albumin. Immunodetection was conducted using a cocktail of mouse anti-PrP monoclonal antibodies: P4 (R-Biopharm AG, Darmstadt, Germany), which targets to amino acids 89-104 of the ovine prion protein sequence [18], at 1:10 000 dilution (0.1 μg/mL) and 6H4 (Prionics AG, Switzerland), which targets to amino acids 144-152 of the human prion protein sequence [19], also at a 1:10 000 dilution (0.1 μg/mL). A biotinylated sheep anti-mouse secondary antibody (GE Healthcare, Buckinghamshire, UK) at 0.05 μg/mL and a streptavidin-horseradish peroxidase (HRP) conjugate (GE Healthcare, Buckinghamshire, UK) were used according to the manufacturer's instructions in conjunction with a chemifluorescent detection system (ECL Plus detection system, GE Healthcare, Buckinghamshire, UK) and imaged using a multimode scanner (Typhoon Imaging System, GE Healthcare, Buckinghamshire, UK). Primary antibody incubations were conducted with the membrane either at room temperature for 1 h or 4°C overnight (≥ 12 h). Secondary antibody and streptavidin-HRP conjugate incubations were conducted at room temperature for 1 h.

Prion protein amino acid sequences were predicted using previously described DNA sequencing methods [20] with slight modifications. Briefly, DNA was extracted from frozen brain or spleen using a commercial kit (High Pure PCR Template Preparation Kit, Roche, Indianapolis, IN, USA). PCR amplification was performed using a primer pair specific for the functional gene (forward primer 223 5'-ACACCCTCTTTATTTTGCAG-3' in intron 2 and reverse primer 224 5'-AGAAGATAATGAAAACAGGAAG-3'), yielding an 830 bp product; and a primer pair specific for the prion precursor protein pseudogene (PRNPψ) (forward primer 379 5'-AAGAAAATTCCTGAGAGAGCAT-3' containing the pseudogene flanking repeat sequence and the adjacent 8 bp from exon 1 and reverse primer 224). Amplification of DNA from deer with PRNPψ yielded a product of 950-974 bp with primers 379/224 depending on the octapeptide repeat number. All PCR reactions were as follows: 95°C for 5 min, followed by 30 cycles of denaturation (95°C, 20 s), annealing (54°C, 20 s) and extension (72°C, 60 s) followed by an extension cycle (72°C, 7 min) under standard buffer conditions (Herculase II Fusion DNA Polymerase, Agilent, Santa Clara, CA, USA). PCR products were analyzed on 1% agarose and visualized with GelRed DNA stain (GelRed, Phoenix Research, Candler, NC, USA). PRNPψ amplification reactions were scored for the presence or absence of a pseudogene-specific band. PCR products were purified by filtration (Millipore Corporation, Billerica, MA, USA) to remove unincorporated dNTPs and primers, then sequenced (Applied Biosystems 3100 genetic analyzer, Carlsbad, CA, USA) with Big Dye Terminator chemistry (PE-Applied Biosystems, Carlsbad, CA, USA) using primer sets described previously [21] CWD-13 (5'-TTTTGCAGATAAGTC ATCATGGTGAAA-3'), CWD-161 (5'-AGGGAAGTCCTGGAGGCAACCGCTATCC-3'), CWD-418 (5'-CACCAAGGCCCCCTACCACTGCTCCAGC-3') and CWD-LA (5'-AGAAGATAATGAAAACAGGAAGGTTGC-3'). The nucleotide numbering for *Prnp *sequence is based on the GenBank cervid sequence (accession no. AF156185) and polymorphisms are reported based on this sequence. PRNPψ amplification reactions were scored for the presence or absence of a pseudogene-specific band.

## Results

**Figure 1:**
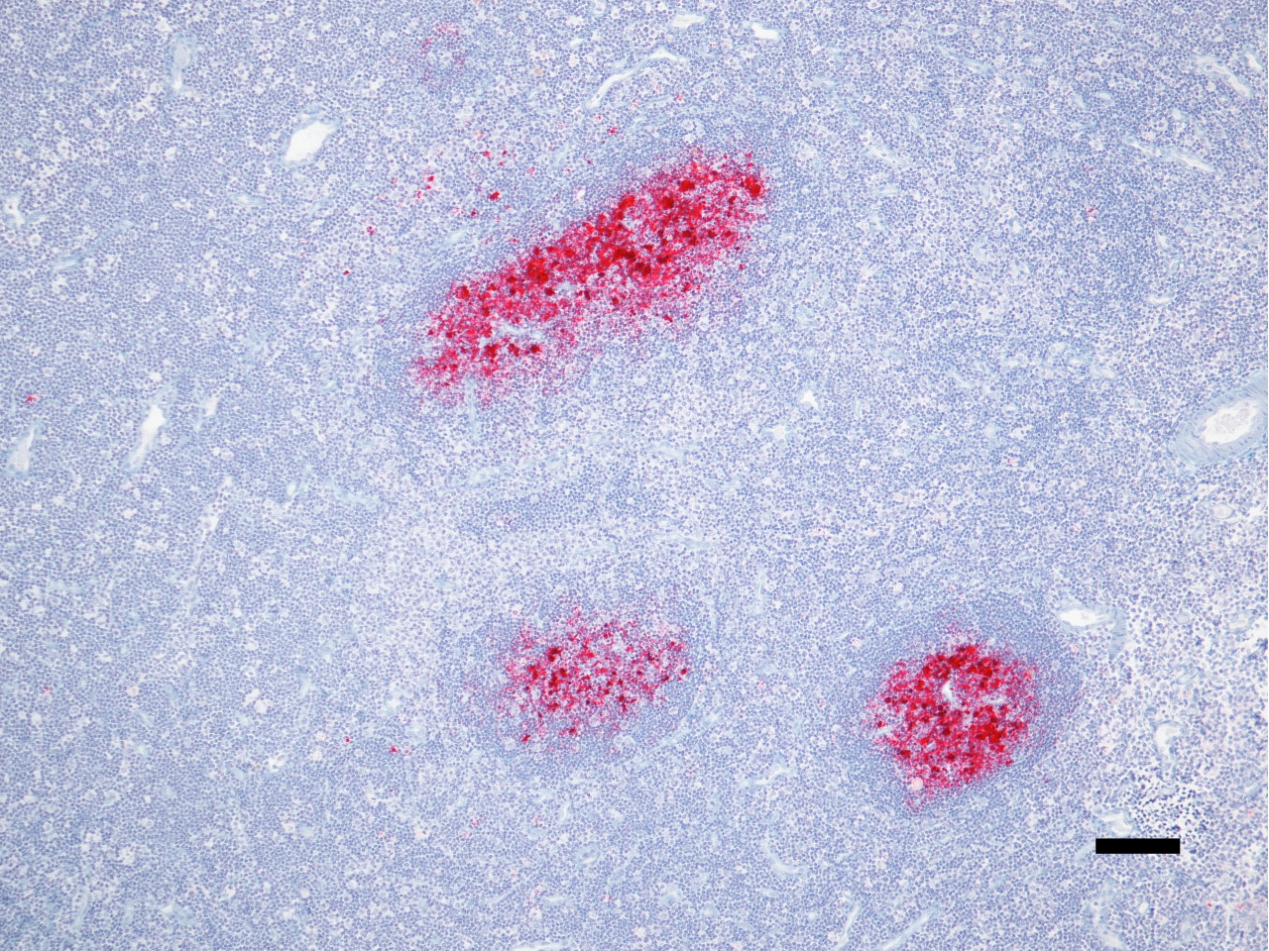
**Immunoreactivity for PrP^Sc ^occurs in lymphoid tissue prior to detection in brain**. Retropharyngeal lymph node from deer 1 was immunoreactive for PrP^Sc ^as early as 6.8 months post-inoculation (pi). Immunoreactivity (red) is intense in the germinal centers of lymphoid follicles. PrP^Sc ^immunohistochemistry with monoclonal antibodies F99/97.6.1 and hematoxylin counterstain. Bar = 100 μm.

Individual results are summarized in Table 1. One animal died at 6.8 months (207 days) post-inoculation (pi) due to intercurrent disease and an abbreviated necropsy was performed. At that time, examination of brain tissue by WB was negative for PrP^Sc^, but IHC of retropharyngeal lymph node and multiple hemal nodes from adjacent to the thoracic aorta were positive (see Figure 1). Lymphoid follicles in the spleen were not immunoreactive for PrP^Sc ^at this early timepoint.

**Table 1:** Summary of findings in white-tailed deer inoculated with the scrapie agent from sheep.

Animal ID	Survival period*	Clinical signs	Spongiform change	WB	IHC obex	IHC cerebellum	IHC cerebrum	IHC retina	IHC lymphoid
1	6.8	-	-	-	-	-	-	-	+

2	15.4	-	-	+	+	-	+	+	+

3	20.4	+	+	+	+	+	+	+	+

4	21.6	+	+	+	+	+	+	+	+

5	22.5	+	+	+	+	+	+	+	+

6	NA	-	-	-	-	-	-	-	-

*post-inoculation time in months; WB = PrP^Sc ^Western blot; IHC = immunohistochemistry for PrP^Sc^; NA = not applicable.

**Figure 2:**
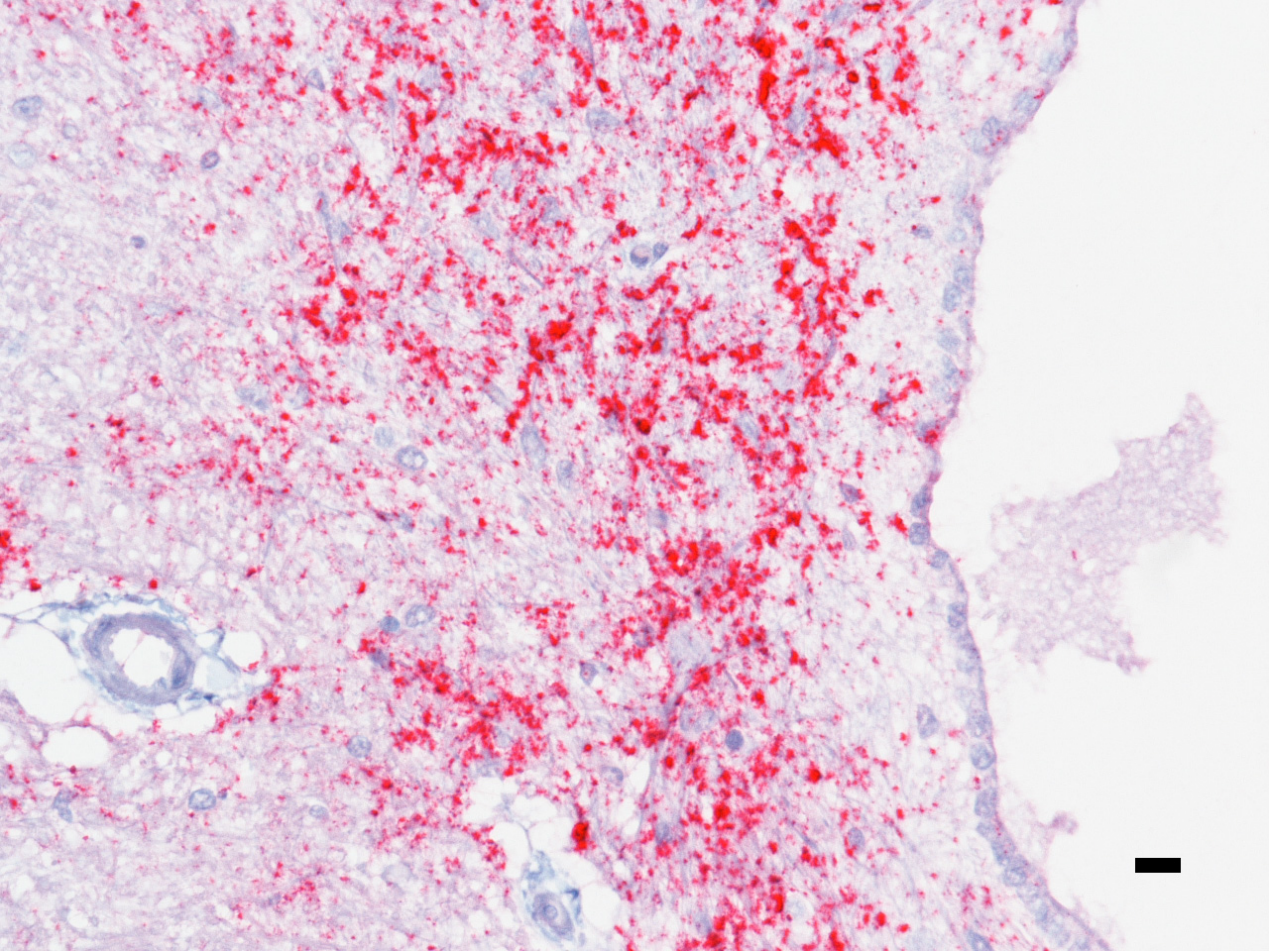
**Brain is immunoreactive for PrP^Sc ^at 15.4 months pi. PrP^Sc ^immunoreactivity is present in brainstem of deer 2 just caudal to the obex**. IR is most intense adjacent to the central canal rather than in the brainstem nuclei. PrP^Sc ^immunohistochemistry with monoclonal antibodies F99/97.6.1 and hematoxylin counterstain. Bar = 10 μm.

Deer necropsied at 15-22 months pi were positive for PrP^Sc ^in brain by WB and in multiple tissues by IHC. Intensity of immunoreactivity (IR) increased with incubation time. Deer necropsied at 15.4 months pi had mild IR in the medulla oblongata at the level of obex that was associated with the central canal (see Figure 2). At this time point, there was moderate IR spanning from the rostral colliculus to the pons in the areas adjacent to the third ventricle and cerebral aqueduct and a focal area of cortical IR near the olfactory bulb. Within the spinal cord, IR was present in small granular to globular aggregrates in the gray matter neuropil adjacent to the central canal and in a perineuronal pattern in the ventral horn. Immunoreactivity in the retina was scant and multifocal being most evident within the cytoplasm of retinal ganglion cells and in loose aggregates within the inner plexiform layer. Lesser IR was present within the outer plexiform layer and surrounding individual nuclei in the inner nuclear layer.

**Figure 3:**
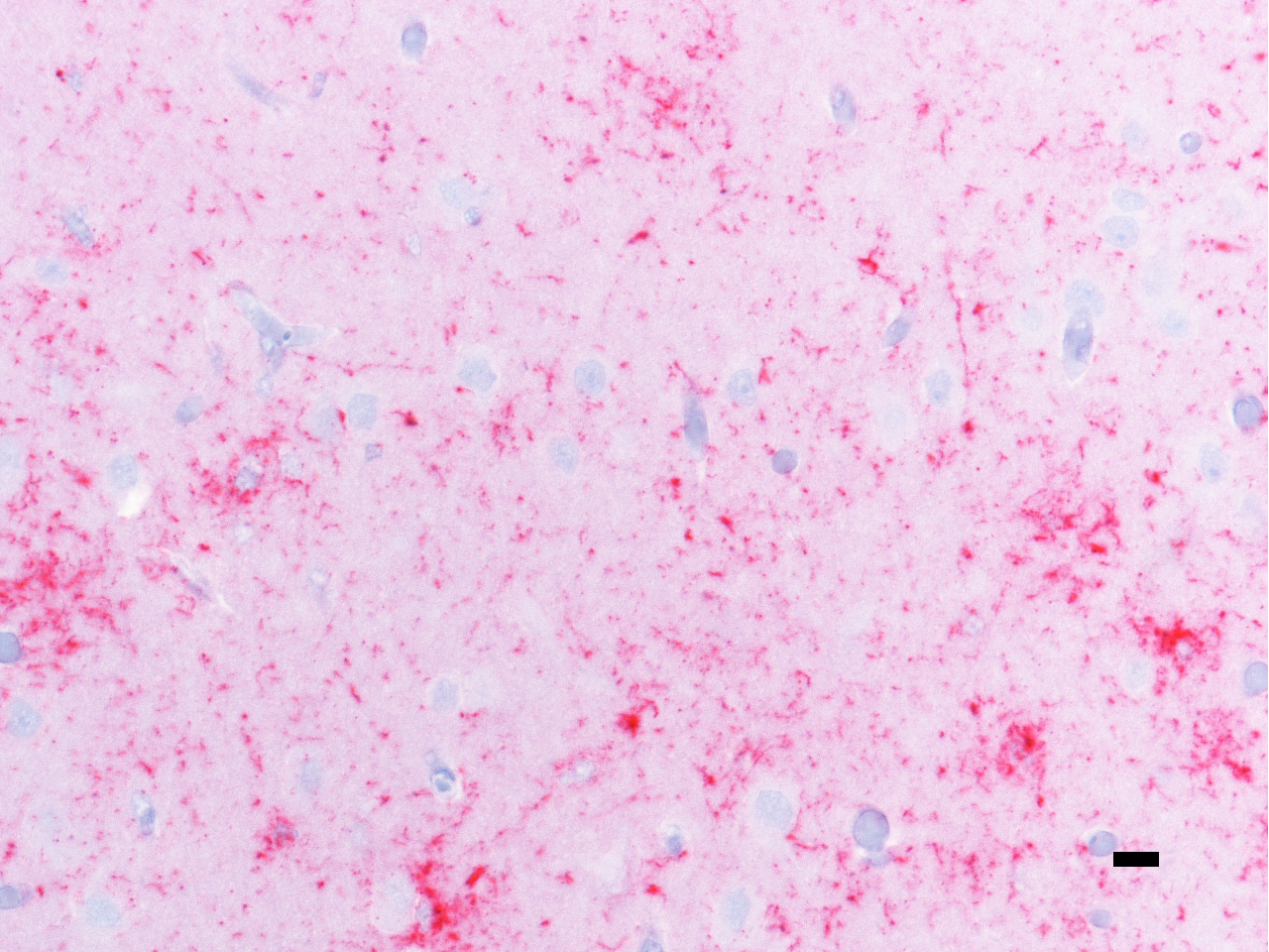
**PrP^Sc ^immunoreactivity is widespread throughout the cerebral cortex in deer with clinical signs of scrapie**. Within the cortex, course granular staining is abundant throughout the neuropil and frequently coalesces in a glial pattern (deer 3). PrP^Sc ^immunohistochemistry with monoclonal antibodies F99/97.6.1 and hematoxylin counterstain. Bar = 10 μm.

Clinical signs included depression and weight loss that progressed to wasting in deer that survived to at least 20 months pi. In one case, sudden death was associated with acute pneumonia. In these deer, tissues with PrP^Sc ^IR by IHC included brain (at levels of the cerebrum, hippocampus, colliculus, cerebellum, and brainstem), trigeminal ganglion, neurohypophysis, retina, spinal cord, and various lymphoid tissues including tonsil, retropharyngeal and mesenteric lymph nodes, Peyer's patches, and spleen. Immunoreactivity was intense in the neuropil of the cerebral cortex (see Figure 3) and was evident regardless of the section examined. Adjacent to the olfactory bulb, the deep cortical lamina, and periventricular areas were most severely affected. Within the brainstem, IR was intense and widely distributed through all but the lateral reaches including the spinocerebellar tract. Further, staining intensity and distribution expanded in the spinal cord to include the entirety of the gray matter. Retina of scrapie-affected deer had marked granular and globular IR in the inner plexiform layer and within the cytoplasm of retinal ganglion cells and scant globular staining around multifocal nuclei in the inner nuclear layer and multifocally within the outer plexiform layer. In these deer, IR was least prominent in the cerebellum where granular to globular IR was present multifocally throughout the molecular layer and was slightly more intense in the granular layer where it occasionally coalesced into loose, multifocal aggregates.

**Figure 4:**
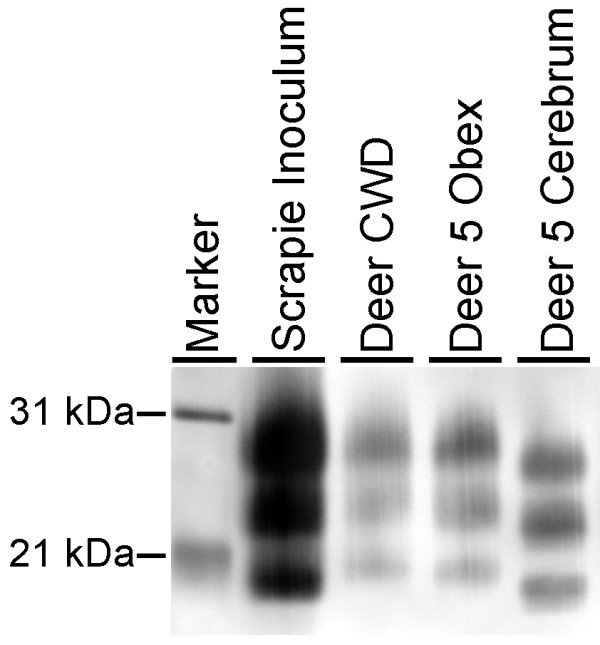
**Representative western blot of deer with scrapie**. This blot compares brain homogenates from the sheep scrapie inoculum (lane 2), a white-tailed deer with CWD [16] (lane 3), and white-tailed deer with scrapie (deer 5, lane 4-5). All brain homogenates are positive using monoclonal antibodies P4 and 6H4 and tissues with a characteristic 3-band pattern representing diglycosylated, monoglycosylated, and unglycosylated forms of PrP^Sc^. Homogenates derived from cerebrum (lane 5) have a lower migration of the unglycosylated band than those derived from brainstem at the level of obex (lanes 4). Lanes were loaded as follows: sheep scrapie inoculums = 0.1 mg, white-tailed deer CWD = 0.175 mg; deer 5 obex = 1 mg, deer 5 cerebrum = 1 mg, Marker = molecular weight marker.

Western blots for the protease resistant fragment of PrP^Sc ^indicated that despite using a single inoculum source, two potential molecular profiles occurred depending on the neuroanatomic location assessed (see Figure 4). In all cases, samples from obex revealed a higher molecular weight than samples from cerebrum. The molecular profile obtained from cerebrum had an unglycosylated band with maximal intensity at approximately 19 kDa and was similar to the scrapie inoculum, but the unglycosylated band obtained from obex samples had a slightly higher electrophoretic mobility similar to that of white-tailed deer intracerebrally inoculated with CWD [16].

**Figure 5:**
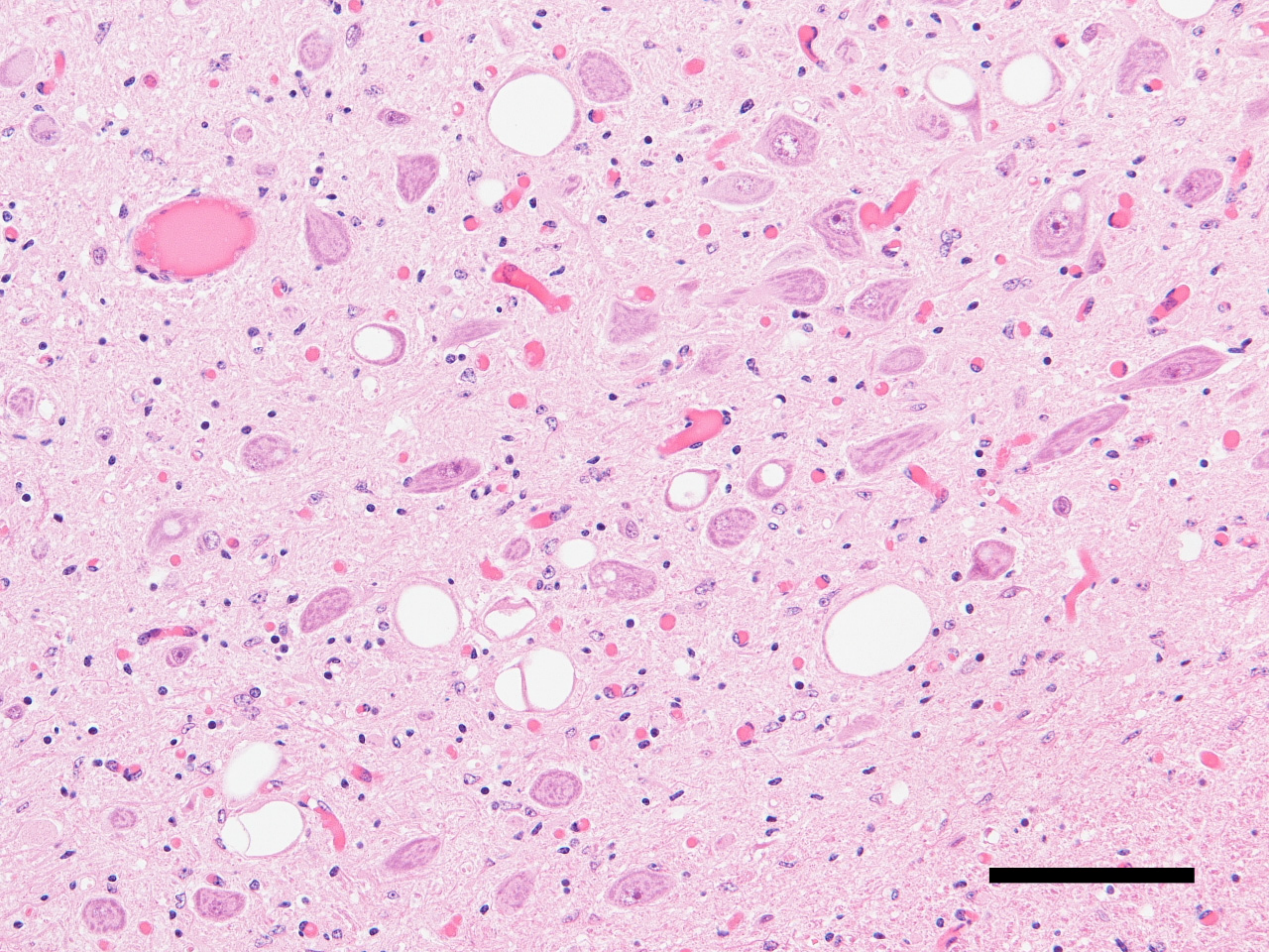
**Deer with scrapie exhibit spongiform change (SE)**. SE was severe in the obex of deer clinically-affected with scrapie (deer 3). Parasympathetic nucleus of the vagus. Hematoxylin and eosin stain. Bar = 100 μm.

**Figure 6:**
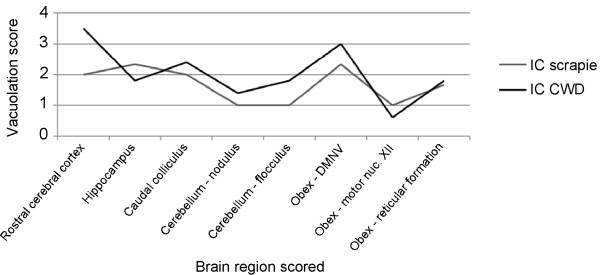
**Gray matter vacuolation profiles for white-tailed deer inoculated intracerebrally with scrapie or CWD**. Vacuolation scores were higher in the cerebral cortex and colliculus and lower in the hippocampus in CWD-affected deer. The converse was present in scrapie-affected deer. Abbreviations: IC, intracerebral; CWD, chronic wasting disease; DMNV, dorsal motor nucleus of the vagus nerve.

Spongiform change was present in the brains of all clinically ill deer, but not preclinical deer with PrP^Sc ^immunoreactivity. In deer with spongiform change, the most severe vacuolation occurred in the piriform cortex, caudate nucleus, thalamic nuclei, and obex (see Figure 5). Vacuolation scores were compiled from 8 gray matter and 2 white matter regions of brain and compared between scrapie- and CWD-affected deer [16] (see Figure 6).

Similar to previous publications [12,20,22], polymorphisms in *Prnp *at codon 96 (G → S) were noted and silent changes were present at codons 146 (aat/aac) and 185 (atc/att). The amino acids present at codons 65, 95, 96, 116, 138, 146, 185, and 226 are shown in Table 2. All other codons were the same as the published cervid sequence (Genbank accession #AF156185).

**Table 2:** Deer diploid genotypes in relation to published reference sequence (Genbank Accession #AF156185).

Codon	65	95	96	116	138	146	185	226	PRNPψ
Animal ID									

Ref	GG	QQ	GG	AA	SS	NN	II	QQ	NE

1	GG	QQ	GS	AA	SS	NN*	II*	QQ	-

2	GG	QQ	GS	AA	SS	NN*	II*	QQ	+

3	GG	QQ	SS	AA	SS	NN*	II*	QQ	-

4	GG	QQ	GS	AA	SS	NN*	II*	QQ	-

5	GG	QQ	SS	AA	SS	NN*	II*	QQ	-

6	GG	QQ	SS	AA	SS	NN*	II*	QQ	-

Polymorphisms were present at codon 96 including GS and SS genotypes, but other loci were the same as the published consensus sequence.

PRNPψ denotes the prion precursor protein pseudogene; Ref = Genbank AF156185.

A = alanine, G = Glycine, I = isoleucine, N = asparagine, S = serine, Q = glutamine, NE: not examined.

*Denotes silent change aat/aac at codon 146 and atc/att at codon 185.

## Discussion

In the interest of further defining the potential origins of CWD, we inoculated deer intracerebrally with a US scrapie isolate. All inoculated deer had evidence of PrP^Sc ^accumulation and those necropsied after 20 months pi (3/5) had clinical signs, spongiform encephalopathy, and widespread distribution of PrP^Sc ^in neural and lymphoid tissues. A single deer that was necropsied at 15.6 months (469 days) pi did not have clinical signs, but had widespread distribution of PrP^Sc^. This highlights the facts that 1) prior to the onset of clinical signs PrP^Sc ^is widely distributed in the CNS and lymphoid tissues and 2) currently used diagnostic methods are sufficient to detect PrP^Sc ^prior to the onset of clinical signs.

In previous studies, intracerebral inoculations of the agent of scrapie in elk caused a disease similar to scrapie and indistinguishable from CWD by microscopic exam or immunohistochemistry [13,14]. However, several elements in that study suggest there are differences between CWD in elk and scrapie in elk. First, there was no lymphoid spread of scrapie in elk. Secondly, no cases were positive in the first 2 years post-inoculation, which was not the case when elk were inoculated with the agent of CWD [23]. Finally, the resultant WBs were indiscernible from the original sheep scrapie inoculum, which has a lower migration pattern of the unglycosylated band when compared to CWD.

In contrast to the study of scrapie in elk, the results of this study suggest that there are many similarities in the manifestation of CWD and scrapie in white-tailed deer. The early and widespread presence of PrP^Sc ^in lymphoid tissues is similar to CWD [24] and different than scrapie in elk. The white-tailed deer in this study were euthanized when unequivocal signs consistent with CWD were present rather than allowing them to progress to advanced disease, but clinical signs of depression and weight loss progressing to wasting were consistent with those described in our previous experiments with CWD [16]. Further, the incubation time of 21-23 months was similar to white-tailed deer of similar genotypes inoculated with CWD in that study. Moreover, western blots done on brain material from the obex region have a molecular profile consistent with CWD and distinct from tissues of the cerebrum or the scrapie inoculum.

The phenomenon describing the interaction of a prion agent with a potential host is referred to as species barrier and explains the ability of a given agent to infect some species and not others [25-27]. The species barrier can manifest as lack of susceptibility, incomplete attack rates, or prolonged incubation times, even in closely related species. Primary passage is usually not efficient between species and sequential passages are required for a TSE strain to stabilize in a new species [26]. The major influence on species barrier is amino acid sequence differences between the donor and recipient hosts [27,28] and the effect on prion protein structure and folding [29]. Results of microscopic and IHC examination indicate that there are differences between the lesions expected in CWD [9,16,30] and those in the deer in this study: amyloid plaques were not noted in any sections of brain examined from these deer and the pattern of immunoreactivity by IHC was diffuse rather than plaque-like and florid plaques were not noted. Vacuolation scores were compiled from 8 gray matter and 2 white matter regions of brain and compared between scrapie- and CWD-affected deer (Figure 6). Average gray matter scores were higher in 6/8 brain regions of CWD- versus scrapie-affected deer, especially rostral cerebral cortex. Additionally, definitive spongiform change was noted in the Purkinje cell, molecular, and granule cell layers of the cerebellum of most CWD-affected deer, but not scrapie-affected deer. Average scores of CWD and scrapie deer trended similarly in the cerebellum and caudal brainstem, but differed among the rostral cerebral cortex, hippocampus, and caudal colliculus. Vacuolation scores were higher in the cerebral cortex and colliculus and lower in the hippocampus in CWD-affected deer. The converse was present in scrapie-affected deer. White matter regions evaluated in both CWD and scrapie deer exhibited no definitive spongiform change or occasional inconclusive axonal vacuolation (data not shown). Despite these differences, the results suggest that the species barrier between sheep and white-tailed deer is relatively weak.

It was remarkable that two western blot patterns occurred in affected deer. When tissues from the obex specifically were assayed by western blot, the molecular profile was consistent with CWD. Western blots performed on samples of cerebrum gave a profile similar to the original sheep scrapie inoculum. When the same regions were analyzed on archived tissues from the study of scrapie in elk [13], only a single WB profile with was evident regardless of region of the brain sampled (data not shown). Possible explanations for the divergent WB profiles are that 1) the source inoculum contained two different sources of scrapie, 2) PrP^Sc ^can be processed differently in different regions of the white-tailed deer brain, or 3) this transmission resulted in the creation of a new TSE strain that occurred concurrently with replication of the original scrapie inoculum. In most instances, the western blot profile obtained in the recipient host is expected to be similar to that of the donor [31] as was the case in cerebrum where the migration pattern was similar to sheep with scrapie. The second migration pattern was consistent with CWD in deer. Co-existence of distinct PrP^Sc ^isoforms can occur in human TSEs [32] where it is associated with distinct neuropathologic profiles [33]. It is known that prion isolates, even those subjected to biological cloning, can be heterogeneous at the molecular level, which allows further selection or adaptation in new hosts [34,35]. Perhaps the presence of two molecular profiles in WB from these deer represents the replication of both the original scrapie isolate favored for replication in sheep and a minor strain not favored in the sheep from which the inoculum was derived, but still present in low levels. Previous work evaluating the effect of genotype on manifestation of CWD in elk demonstrated that homozygosity for leucine at residue 132 (132LL) was associated with a novel folding pattern resulting in a shift of the proteinase K cleavage site when compared with tissues from elk with 132 MM or 132LM genotypes [36]. It remains to be determined whether the findings in 132LL elk represent replication of a minor strain or alternative processing by elk with that genotype. The significance of the two distinct western blot profiles found here will be explored in future studies that will ascertain whether the molecular profiles are conserved upon subpassage.

In this study, there were amino acid polymorphisms at codon 96 that may have affected incubation times, but genotype is an unlikely explanation for the divergent western blot patterns as the difference in profile noted in cerebrum and obex was noted in all deer regardless of genotype. Previous studies suggest that deer with serine (S) rather than glycine (G) at locus 96 in the prion protein were less likely to be found in the CWD-infected population. Susceptibility in this study did not appear to be associated with polymorphisms at codon 96, but intracerebral inoculation could be responsible for bypassing contributions to genetic susceptibility due to peripheral factors [37]. The deer used in this study may not be an accurate proportional representation of genotypes present in the wild population as we had a higher than expected rate of 96S genotypes. The results of our work and the studies of CWD in white-tailed deer [38] suggest that free ranging white-tailed deer in general may be more susceptible to sheep scrapie than the deer used in our experiment due to the predominance of the 96G codon. A recent report of oral transmission of CWD to white-tailed deer similarly describes different western blot patterns that were genotype related [38], but study specific differences including differences in genotypes assessed, source inoculum, and assessment of western blots derived from whole brain rather than region specific homogenates may preclude further comparison of that study with results presented here.

It is possible that biochemical differences between brain regions were the cause of the unexpected molecular profile although usually this results in variances in the glycosylation patterns rather than molecular weight [11]. Provocative work in mice expressing the cervid prion protein suggests that glutamine at codon 226 of the prion protein, similar to the deer in this study, may confer a lack of stability that results in unstable strain propagation of CWD [39]. One major difference between the findings in these deer and those in the mouse study is that the western blot profile of the mouse strains was identical whereas phenotypic differences allowing strain differentiation were noted on neuropathology. Further passage is required in deer or transgenic mice to determine if the different molecular profiles are associated with distinct disease phenotypes. Additional passage of brain material derived from the regions with divergent molecular profiles is underway to determine if passage into deer or transgenic mice results in a stable propagation of these isolates. These transmission studies in conjunction with detailed analysis of biochemical characteristics of the resultant PrP^Sc ^will allow a better assessment of whether natural transmission of sheep scrapie to white-tailed deer presents a real risk and whether these results indicated the potential for selection or creation of new TSE strains after interspecies transmission.

It is unlikely that CWD will be eradicated from free-ranging cervids, and the disease is likely to continue to spread geographically [10]. However, the potential that white-tailed deer may be susceptible to sheep scrapie by a natural route presents an additional confounding factor to halting the spread of CWD. This leads to the additional speculations that 1) infected deer could serve as a reservoir to infect sheep with scrapie offering challenges to scrapie eradication efforts and 2) CWD spread need not remain geographically confined to current endemic areas, but could occur anywhere that sheep with scrapie and susceptible cervids cohabitate.

This work demonstrates for the first time that white-tailed deer are susceptible to sheep scrapie by intracerebral inoculation with a high attack rate and that the disease that results has similarities to CWD. These experiments will be repeated with a more natural route of inoculation to determine the likelihood of the potential transmission of sheep scrapie to white-tailed deer. If scrapie were to occur in white-tailed deer, results of this study indicate that it would be detected as a TSE, but may be difficult to differentiate from CWD without in-depth biochemical analysis.

## List of Abbreviations

A: alanine; CWD: chronic wasting disease; G: glycine; I: isoleucine; IR: immunoreactivity; L: lysine; M: methionine; N: asparagine; NE: not examined; IHC: immunohistochemistry; PI: post inoculation; *Prnp*: prion protein gene; PRNPψ: prion precursor protein pseudogene; S: serine; Q: glutamine; SE: spongiform encephalopathy; TSE: transmissible spongiform encephalopathy; WB: western blot; WTD: white-tailed deer

## Competing interests

The authors declare that they have no competing interests.

## Authors' contributions

JJG and JDS carried out experimental work (inoculations, sample acquisition), interpreted the pathologic findings (histopathology, immunohistochemistry, western blot analysis), and drafted the manuscript. RAK contributed to the design of the experimental study and provided technical advice concerning immunohistochemistry. All authors read and approved the final manuscript.
